# Self-buffered epitaxy of barium titanate on oxide insulators enables high-performance electro-optic modulators

**DOI:** 10.1038/s41377-025-02081-9

**Published:** 2026-01-02

**Authors:** Chenguang Deng, Yutong He, Wenfeng Yang, Han Yu, Zijian Hong, Hao Liu, Haojie Han, Wei Li, Yunpeng Ma, Zhongshan Zhang, Yongjun Wu, Jing Ma, Bing Xiong, Changzheng Sun, Rong Yu, Jing-Feng Li, Ji Zhou, Yi Luo, Qian Li

**Affiliations:** 1 State Key Laboratory of New Ceramic Materials, School of Materials Science and Engineering, Tsinghua University, Beijing, China; 2 Beijing National Research Centre for Information Science and Technology (BNRist), State Key Laboratory of Space Network and Communications, Department of Electronic Engineering, Tsinghua University, Beijing, China; 3 State Key Laboratory of Silicon and Advanced Semiconductor Materials, School of Materials Science and Engineering, Zhejiang University, Hangzhou, Zhejiang China; 4 Zhejiang Key Laboratory of Advanced Solid State Energy Storage Technology and Applications, Taizhou Institute of Zhejiang University, Taizhou, Zhejiang China; 5 Institute of Fundamental and Transdisciplinary Research, Zhejiang University, Hangzhou, China; 6 Beijing National Laboratory for Condensed Matter Physics, Institute of Physics, Chinese Academy of Sciences, Beijing, China

**Keywords:** Optical materials and structures, Other photonics

## Abstract

Integrated photonics has emerged as a promising alternative for data communication and computing, ferroelectric BaTiO_3_ (BTO) stands out for its exceptional electro-optic response among candidate materials. However, direct epitaxial growth of BTO entails a fundamental trade-off: substrates with low refractive index are required for strong optical confinement, yet those with large lattice mismatch degrade film crystalline quality and electro-optic performance. We report a buffer-free, strain-engineered approach to integrate high-performance BTO thin films directly on LaAlO_3_-Sr_2_TaAlO_6_ (LSAT) oxide-insulator substrates. By exploiting a self-buffer layer formed during the initial growth stage, we achieve periodic in-plane strain modulation that stabilizes a polymorphic phase boundary with orthorhombic polar nanoregions, yielding a Pockels coefficient exceeding 358 pm V⁻¹ and a Curie temperature raised to 200 °C. Leveraging this material platform, we demonstrate the first realization of a Mach–Zehnder modulator using epitaxial BTO on LSAT. The device exhibits a half-wave voltage–length product of 0.7 V cm at 1550 nm, which closely matches finite-element simulations, and supports a 6-dB electro-optic bandwidth of 28 GHz. Our results validate BTO on LSAT as a viable photonic platform for scalable, low-voltage and high-speed modulators.

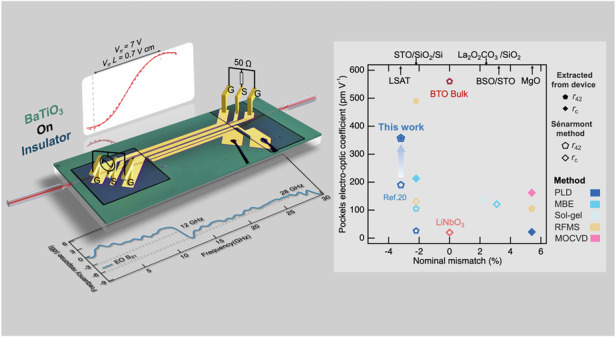

## Abstract (web summary)

Epitaxial BaTiO_3_ on LSAT shows large Pockels coefficients via polymorphic phase boundaries, enabling low-voltage, high-speed electro-optic modulators.

## Introduction

As transistor miniaturization approaching fundamental physical limits, integrated photonics has gained increasing attention as an emerging alternative in recent years^1^. In data communication, integrated photonic platforms offer inherent advantages such as high bandwidth and low transmission loss, where high-performance electro-optic (EO) modulators are essential components^2,3^. Although silicon photonic modulators based on the plasmonic dispersion effect have been developed, their modulation efficiency and speed remain limited^4^. Thin-film LiNbO_3_ has also attracted considerable interest^5,6^ yet its production relies on complex and expensive ion slicing and bonding processes^7^. Moreover, the intrinsic low EO coefficients of both silicon and LiNbO_3_ further limit their suitability for dense photonic integration. In contrast, ferroelectric perovskite BaTiO_3_ (BTO) has recently emerged as a promising candidate for integrated photonics due to its outstanding EO response^8,9^. Significant progress has been made in employing BTO thin films for EO modulation with multi-functionality, including cryogenic-temperature modulation^10^ and non-volatile photonic phase shifting^11^. Although Pockels EO coefficients ranging up to an order of magnitude higher than that of LiNbO_3_ have been reported, they remain significantly lower than the bulk value^12^. Direct bonding of BTO to SiO_2_/Si wafer can partially restore its intrinsic EO response^13^, yet developing simple and cost-effective epitaxial growth strategies on oxide insulator platforms remains essential for realizing high-performance and scalable BTO-based devices.

Addressing these challenges requires not only suitable growth methods but also careful selection of low-refractive-index substrates that support strong optical confinement. Although SrTiO_3_ (*n* = 2.28 at 1550 nm) substrates allow high-quality BTO film growth, its comparable refractive index with BTO (*n* = 2.26 at 1550 nm) severely limits optical confinement within the films. MgO (*n* = 1.7 at 1550 nm) provides a large refractive index contrast, but the severe lattice mismatch with BTO leads to poor crystallinity and degraded EO performance^14–16^. (LaAlO_3_)_0.3_-(Sr_2_TaAlO_6_)_0.7_ (LSAT, *n* = 1.99 at 1550 nm) potentially offers a more balanced trade-off, with improved lattice compatibility and better index mismatch. Large-size (up to 3 inch) LSAT wafers are also commercially available at considerably lower cost compared with those of rare-earth scandates^17^. However, compressive strain in BTO films grown on LSAT often induces out-of-plane or mixed polarization which are partially switchable, incompatible with in-plane electrode configurations used in photonic devices^18^. While natural strain relaxation favors in-plane polarization, it frequently degrades crystallinity and increases surface roughness of BTO films^19^, leading to reduced EO efficiency and higher optical losses.

Our previous studies have demonstrated that buffer layers with lattice parameters closely matched to BTO can promote in-plane domain configuration and enhance EO performance^20^. Here, we propose a more advanced, heterogeneous buffer-free approach that leverages intrinsic lattice mismatch to engineer local structural distortions and a multiphase architecture, thereby further enhancing the EO response of BTO films. This strategy is inspired by lead-free ferroelectrics such as (K,Nb)NbO_3_, where multiphase coexistence near the polymorphic phase boundary (PPB) results in polar nanoregions imbued with orthorhombic and tetragonal phases, enabling ultrahigh piezoelectric response^21–23^. Traditionally, such multiphase boundaries are created through intricate compositional modifications. However, comparable boundaries can also be achieved by reducing crystal symmetry via local structural distortions, without the need for complex compositional tuning^24^. A similar hypothesis suggests that domain-wall-associated polar nanoregions, particularly those stabilized by strain-induced structural transitions, play a key role in enhancing the functional properties of the material. This is supported by theoretical simulations, which attribute the enhanced EO response in strained BTO films to the Ising–Néel transition and the emergence of pseudo-orthorhombic phases at 90° domain walls^25^. Our approach thus highlights the potential of strain-engineered domain-wall structures in enabling functional enhancement in ferroelectric thin films.

Building on this strategy, we introduce a self-buffer layer for epitaxial growth of BTO on LSAT, achieving a Pockels coefficient *γ*_42_ exceeding 358 pm V⁻¹ and elevating the Curie temperature from 120 °C (bulk) to 200 °C. Leveraging this high-performance EO platform, we demonstrate the first on-chip BTO electro-optic modulator on an LSAT oxide insulator, with a half-wave voltage length product *V*_π_*L* of 0.7 V cm (1550 nm) and a 6 dB EO bandwidth of ~28 GHz. Crucially, the films are fabricated via a flexible, cost-effective single physical vapor deposition process (as demonstrated here via pulsed-laser deposition), eliminating the need for wafer bonding or complex post-growth treatments. Moreover, our strain-engineered domain structure design can be readily transferred to other emerging ferroelectric perovskites to tune the balance between lattice mismatch and thin-film performance, opening a new pathway to multifunctional integrated photonics.

## Results

### Periodic lateral strain engineering

Epitaxial growth of BTO thin films on lattice-mismatched substrates involves a complex interplay between strain relaxation, domain structure evolution and crystallinity, all of which collectively determine the resulting electro-optic properties^26^. On highly mismatched substrates (e.g., MgO), strain relaxation through various forms of dislocations promotes a three-dimensional island growth mode, compromising the surface morphology and crystallinity and thereby degrading the electro-optic performance^14–16^. In contrast, layer-by-layer or step-flow growth modes preserve epitaxial relationships and yield high-quality films. However, they typically impose compressive strain constraints that tend to stabilize *c*-oriented BTO domains, thereby suppressing in-plane polarization responses that are essential for large electro-optic coefficients^27^.

**Fig. 1:**
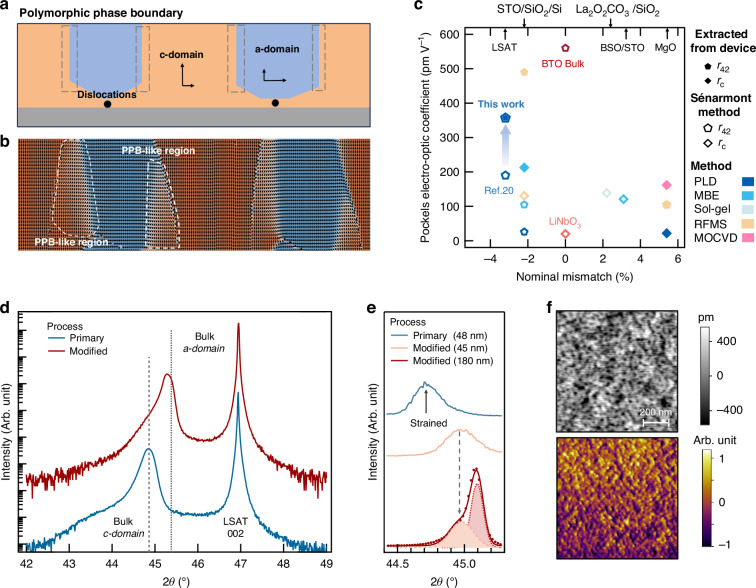
Process-guided structural engineering of BaTiO3 (BTO) thin films for enhanced electro-optic performance via periodic in-plane strain. **a** Schematic of the structural configuration in BTO thin films fabricated using the modified process, in which alternating *a*- and *c*-domains form polymorphic phase boundaries. **b** Phase-field simulation results showing the emergence of orthorhombic phase (O-phase) polar nanoregions at the domain boundaries. Arrows represent the three-dimensional polarization vectors. The background color indicates domain types: brown for *c*-domains and blue for *a*-domains. **c** Comparison of Pockels electro-optic coefficients for BTO films grown on substrates with varying lattice mismatch using different deposition methods. This work achieves a significantly enhanced coefficient of BTO thin films grown on substrates with large compressive mismatch. *r*_c_ denotes the effective EO coefficient, while *r*_42_ is an EO tensor component of BTO films. **d** XRD *θ*–2*θ* scans near the (002) reflection for BTO films grown on LSAT via the primary and modified processes. **e** XRD *θ*–2*θ* scans for films of varying thicknesses and processes, indicating strain relaxation and the formation of a self-buffer layer at the initial stage of deposition in the modified process. **f** (Top) Atomic force microscopy image and (bottom) projected in-plane piezoresponse image of the modified BTO film. The projected piezoresponse is synthesized from both phase and amplitude signals

To address this trade-off, we introduce a strain modulation strategy for periodic structures design, and the schematic diagram of the resulting domain configuration is illustrated in Fig. 1a. At the early stage of film growth on compressive substrates, periodic nucleation sites for dislocations are introduced, allowing localized lattice regions above these sites to undergo strain relaxation and thereby stabilize into *a*-domains. Meanwhile, adjacent regions remain strained and consequently stabilize into *c*-domains. Therefore, this process leads to a laterally periodic *a*/*c* domain configuration. Unlike conventional BTO films where strain relaxation typically completes within a thickness of ~40 nm^28^, this built-in lateral strain variation can be sustained over greater thicknesses through a well-controlled step-flow growth mode. Phase-field simulations (see Fig. S5 and Supplementary Information Note 5) in Fig. 1b further demonstrate that the engineered *a*/*c* domain configuration induces the formation of transitional regions near the domain walls, characterized by polar nanoregions with an orthorhombic (O-) phase. Notably, such structural features bear a strong resemblance to the multiphase coexistence observed near polymorphic phase boundaries (PPBs) in (K,Nb)NbO_3_, where the interplay between tetragonal (T-) and O-phase leads to enhanced dielectric and piezoelectric responses due to facilitated local polarization rotation dynamics^21–24,29^. The effectiveness of our approach is further substantiated by comparing the measured EO coefficients of our BTO films with reported values for films fabricated by different deposition methods on various substrates (Fig. 1c, see Supplementary Information Note 1 for references)^12,15,16,20,30–38^. While BTO films on substrates with severe lattice mismatch often suffer from significant performance degradation, our films exhibit substantially enhanced Pockels coefficients. These results underscore the effectiveness of our strategy in maintaining high crystallinity while introducing functionally beneficial polar nanostructures, thereby overcoming the limitations of conventional strain engineering.

As previously discussed, it is crucial to facilitate early-stage strain relaxation and ensure high crystallinity by tailoring the growth conditions. As presented in Fig. 1d, *θ*–2*θ* X-ray diffraction (XRD) patterns reveal clear distinctions between films grown under the primary and modified processes (see details in Method). The film prepared by the primary process displays a sharp (002) reflection that coincides with the position of *c*-domain in bulk BTO, indicating a compressively strained *c*-domain structure. In contrast, the modified process results in a high-angle shifted (002) peak, suggesting a reduced out-of-plane lattice parameter (~4.0 Å) and a quasi-*a*-domain state through the strain relaxation. To investigate the evolution of strain relaxation during growth, thickness-dependent XRD were performed (Fig. 1e). For the 48 nm-thick film grown by the primary process, the (002) peak posited at lower angles confirms a substantial out-of-plane lattice expansion due to the compressive strain, in contrast with the 45 nm-thick film grown by the modified process. The high-angle (002) peak of the latter indicates that the strain has largely been relaxed during the initial growth stage. As the film thickness increases to 180 nm, the diffraction peak remains sharp with only a minor shoulder. This suggests that a relaxed interfacial layer formed during the early stage acts as a self-buffer, enabling subsequent high-quality epitaxial growth with minimal strain accumulation, and maintains the overall high crystallinity with a rocking curve width of 0.072° (shown in Fig. S1). The reciprocal space maps (RSMs) shown in Fig. S2 further support the presence of a mixed-domain structure. The mixed *a*- and *c*-domains lead to a diffused profile of the BTO (103) reflection, indicating the coexistence of multiple strain states stabilized by the modified growth process.

Topographic and piezoresponse force microscopy (PFM) images presented in Fig. 1f confirm the excellent crystalline quality and characteristic domain configuration of the film. The 180 nm-thick film exhibits atomically smooth surfaces with a root-mean-square roughness of 0.2 nm. The projected piezoresponse image reveals curvy and diffused domain boundaries, in contrast to the sharply defined patterns typical of conventional ferroelectrics, indicating the presence of complex polarization distribution underlain by the *a*/*c* domains and intermediate O-phase nanoregions.

**Fig. 2:**
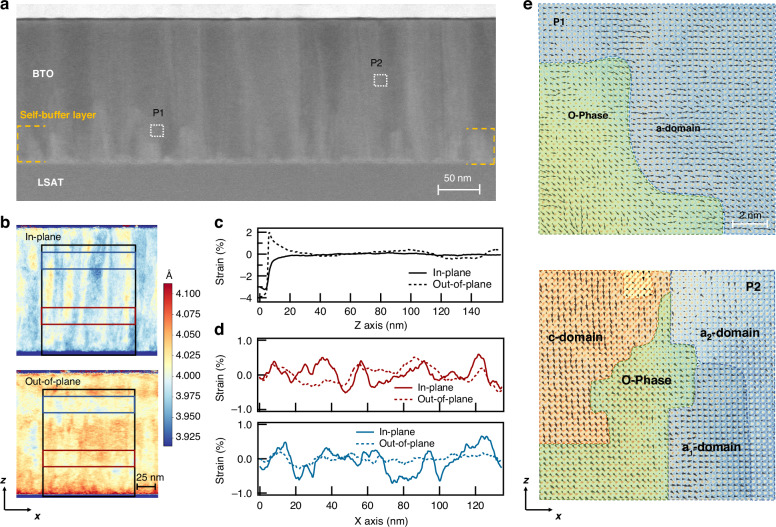
Microscopic analysis of the strain and polarization states in self-buffered BTO thin films. **a** Cross-sectional low-magnification STEM image of the BTO film. A ~40 nm-thick self-buffer layer is observed at the bottom, distinct from the upper region exhibiting lateral contrast modulations. **b** Strain maps for the in-plane (up) and out-of-plane (down) components, reconstructed from the NED results. **c** Strain profiles of the average in-plane and out-of-plane strain components along the growth z (<001>) direction. **d** Lateral strain profiles extracted at different depths, confirming the emergence of periodic in-plane strain modulations above the buffer layer. The line color corresponds to the regions selected in **b**. **e** Atomic-resolution HAADF-STEM images for two representative regions, showing (up) O-phase regions near the self-buffer layer and (down) complex polar structures at a domain boundary. The reconstructed polarization vectors are overlaid

Figure 2 presents scanning transmission electron microscopy (STEM) results for the self-buffered BTO films, revealing the evolution of lattice modulations along both the growth direction and the in-plane axis. The low-magnification high-angle annular dark-field (HAADF-STEM) image (Fig. 2a) displays a well-defined bilayer structure. A ~40 nm-thick bottom region exhibits distinct contrast features compared to the upper layer, identified as the self-buffer layer. Fig. S3 shows a sharp atomic interface between the film and substrate, where the ~3% lattice mismatch between LSAT and BTO is relieved by periodic edge dislocations (approximately one every 15 nm), as expected for mismatch compensation. The upper part of the film exhibits pronounced stripe-like lateral modulations. These periodic contrast variations, oriented along the in-plane direction, suggest the emergence of a spatially ordered lattice distortion (see Fig. S3 and Supplementary Information Note 2).

To further quantify the periodic structural modulations, nanobeam electron diffraction (NED) was performed, as depicted in Fig. 2b. The region above the buffer layer reveals well-defined stripe-like contrast along the in-plane direction, indicative of a periodic in-plane strain modulation that persists throughout the remaining film thickness. The out-of-plane strain component (*ε*_zz_) displays a markedly different behavior. Near the substrate interface, a pronounced lattice expansion is observed, consistent with strong out-of-plane strain imposed by substrate clamping. However, the *ε*_zz_ component becomes laterally uniform above the self-buffer layer, showing little variation along the x direction and lacking the periodic features observed the in-plane strain component (*ε*_xx_). Statistical analysis of the average lattice parameters within selected boxed regions confirms a clear transition across the self-buffer boundary. The in-plane and out-of-plane lattice constants show significant disparity below ~40 nm, whereas they gradually converge above this boundary (Fig. 2c), consistent with the visual boundary of the self-buffer region. Furthermore, line profiles extracted along the in-plane direction at various depths consistently show periodic variations in the in-plane lattice parameters (Fig. 2d).

To establish the correlation between the strain modulation and polarization distribution, local polarization vectors were extracted from atomically resolved HAADF-STEM images. Figure 2e presents atomic-resolution images acquired from representative regions near the self-buffer layer (P1) and *c*/*a*-domain boundaries (P2). In the P1 region, strain relaxation near the self-buffer layer induces the formation of *a*-domains accompanied by a high density of O-phase regions polarized along the <101> directions. Meanwhile, the P2 region reveals a more complex polarization distribution, featuring both in-plane and out-of-plane components as well as distinct O-phase polar nanoregions. The polarization vectors in these nanoregions gradually bridge the adjacent *a*- and *c*- domains (T-phase). This intermediate-phase characterizes the presence of local polymorphic phase boundaries and suggests an enhanced polar instability near the domain walls. The emergence of such complex polar textures and domain wall geometries highlights the strong coupling between the lateral strain modulation and polarization response. Notably, the experimentally observed features are in qualitative agreement with the phase-field simulated polarization patterns in Fig. 1b and Fig. S5.

Taken together, the formation of a self-buffer layer relieves the substrate-induced strain, enabling the stabilization of a quasi-periodic lateral strain field in the upper layers. This in-plane strain modulation, in turn, governs the formation and distribution of ferroelectric domains and promotes the emergence of rotationally distorted polar structures near domain walls.

### Electro-optic enhancement via polymorphic nanoregions

**Fig. 3:**
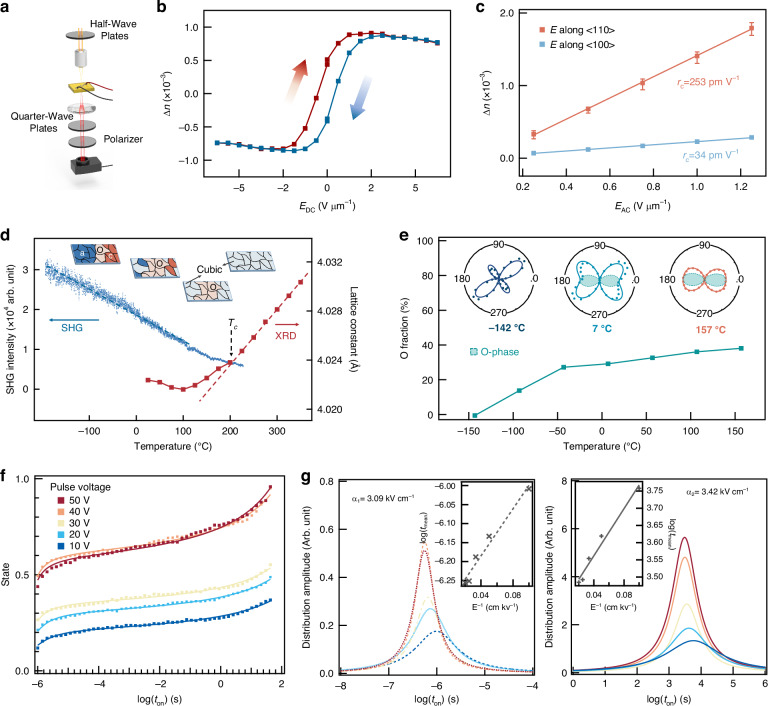
Enhanced electro-optic response in self-buffered BTO films via polymorphic phase nanoregions. **a** Schematic illustration of the optical setup for EO measurements. **b** Changes in the refractive index as a function of DC bias. AC field = 0.625 V μm^−1^. **c** Changes in the refractive index as a function of AC electric field applied along the <110> and <100> in-plane directions. DC field = 3.125 V μm^−1^. The effective EO coefficients are extracted from the slopes. **d** Temperature-dependent evolution of second-harmonic generation (SHG) intensity and out-of-plane lattice constant obtained from XRD. Inset: schematic illustration of domain evolution with temperature, where “a, c, o” denotes T-phase *a*-/*c*-domain and O-phase domain, respectively. **e** Temperature dependence of the O-phase fraction, extracted by fitting the SHG polar patterns. Inset: SHG polarimetry fitting results at representative temperatures, with green-shaded regions indicating the contribution of the O-phase. **f** Normalized EO response measured after application of various numbers of electric pulses superimposed on a 10 V DC bias, revealing ferroelectric domain switching kinetics. The states “0” and “1” correspond to the two extreme EO responses measured in the BTO films poled using negative and positive electric fields, respectively. **g** Domain switching time extracted using a constrained nucleation model, fitted with two-segment Lorentzian distributions. Inset: linear fit used to extract the activation field *α*

We employed a home-designed Sénarmont system (Fig. 3a) to characterize electro-optic performance of the BTO films^39,40^. Under an applied DC bias, the polarization switching dynamics of domains oriented along the <110> direction yield a pronounced EO hysteresis loop (Fig. 3b), consistent with ferroelectric switching behavior. The relatively low coercive field observed is attributed to the presence of polymorphic phase nanoregions, which lower the energetic barrier for in-plane polarization rotation. The EO response measured under AC excitation displays a strong crystallographic anisotropy. As shown in Fig. 3c, a peak effective EO coefficient of *r*_c_ = 253 pm V⁻¹ is obtained when the electric field is applied along the <110> direction, while a significantly lower value of *r*_c_ = 34 pm V⁻¹ is observed under an electric field along the <100 > . This anisotropy arises from the inherent form of the EO tensor of tetragonal BTO. Specifically, the large EO response along the <110> can be ascribed to the projection of the intrinsic *r*_42_ coefficient, which is calculated to be 358 pm V⁻¹ according to Supplementary Information Note 4. Compared with previous studies^20^, this enhancement is attributed to the rational structural design that facilitates the formation of O-phase nanodomains and local PPBs.

To identify the role of intermediate O-phase nanoregions, we performed in-situ temperature-dependent structural characterizations (see Supplementary Information Note 6). As shown in Fig. 3d, second harmonic generation (SHG) intensity decreases almost linearly from −150 °C to 100 °C, without any abrupt discontinuity. Since SHG is highly sensitive to macroscopic lattice symmetry breaking, the absence of abrupt changes suggests that no long-range phase transition occurs within this temperature range^41^. Interestingly, a noticeable dip in the SHG intensity is observed near 120 °C, indicative of local polarization vector rearrangements. Further insights are provided by the temperature-dependent XRD. As shown in Fig. 3d, the out-of-plane lattice constant increases linearly with temperature above 200 °C, characteristic of a paraelectric phase. This temperature region can thus be assigned as the Curie temperature *T*_c_. Below 200 °C, the lattice constant clearly deviates from linear thermal expansion, reflecting the presence of distinct domain responses. From 0 °C to 120 °C, the out-of-plane lattice constant decreases with increasing temperature, consistent with a bulk-like thermal contraction behavior of the mixed *a*- and *c*-domains toward the cubic phase. The thermal expansion recovers a positive trend between 120 °C and 200 °C. Although the elevated *T*_c_ has traditionally been ascribed to epitaxially constrained *c*-domains^42^, it is also necessary to consider the potential influence of polymorphic phase nanoregions. Unlike classical long-range ordered ferroelectric systems, these nanoregions may give rise to spatially localized polar instabilities, thus resulting in a broadened structural transition behavior.

To further elucidate the structural evolution behaviors, we performed temperature-dependent SHG polarimetry (Fig. 3e). By virtue of the symmetry sensitivity of SHG patterns, the O-phase fraction can be extracted by fitting the angular response^43^. As shown in Fig. 3e, the O-phase ratio remains nearly constant from room temperature to 160 °C. This temperature-invariant behavior indicates that the intermediate O-phase nanoregions are structurally robust and thermally stable. These findings corroborate the hypothesis that both the diffuse phase transition and the elevated Curie temperature arise from the persistent polymorphic nanoregions, rather than from an abrupt symmetry breaking. Altogether, the results reveal non-classical ferroelectric behavior in self-buffered BTO films due to the nanoscale structural heterogeneity.

The domain switching kinetics were examined via pulsed EO and SHG mapping measurements under <110> electric fields (see Fig. S4 and Supplementary Information Note 3). The coexistence of *a*-/*c*-domains and O-phase nanoregions provides a continuous pathway for polarization rotation, leading to switching behaviors that deviate from classical phenomenological models such as the nucleation-limited switching (NLS) model^44,45^. As shown in Fig. 3f, the switching kinetics under different pulse voltages can be well described by a superposition of two distinct NLS components. The fast component is attributed to the in-plane switching of *a*-domains, while the slower one likely arises from the in-plane reorientation of *c*-domains. Previous studies showed that the substrate elastic clamping effect in epitaxial films can suppress the polarization reorientation of *c*-domains^46^. However, as shown in Fig. 3g, the Lorentzian distributions of switching time here extracted from the two-stage NLS model reveal that the activation fields (*α*) associated with both the fast and slow components are comparable. This thus indicates a reduction in the switching energy barrier for the *c*-domains, due to the presence of intermediate O-phase nanoregions which facilitate polarization rotation pathways via local O-T structural transitions. Overall, these results highlight the distinctive complex switching kinetics of self-buffered BTO films dictated by the engineered PPBs.

### On-chip electro-optic modulator

**Fig. 4:**
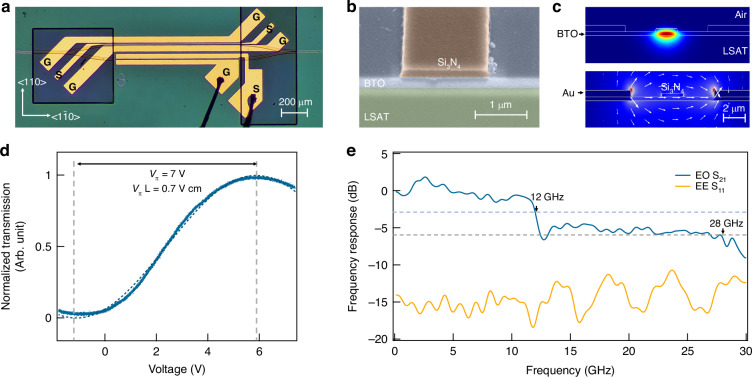
BTO on LSAT as an oxide platform for integrated high-performance electro-optic modulation. **a** Optical micrograph of the fabricated Mach–Zehnder interferometer (MZI) based modulator, implemented on self-buffered BTO film in conjunction with Si_3_N_4_ strip waveguides. Gold electrodes configured in a ground–signal–ground (GSG) layout are used for high-speed driving. **b** Tilted-view scanning electron microscopy (SEM) image of the edge-coupling interface. The brown region corresponds to the Si_3_N_4_ waveguide, the blue region to the BTO film, and the green region to the LSAT substrate. **c** Simulated mode profiles of the photonic TE mode (top) and quasi-static electric field (bottom) within the modulator cross-section. **d** Normalized optical transmission as a function of the applied voltage. The extracted half-wave voltage (*V*_π_) is 7 V, corresponding to a half-wave voltage-length product (*V*_π_*L*) of 0.7 V cm. **e** Frequency response of the MZI modulator, showing the electro-optic bandwidth and the impedance matching condition of the microwave input

To evaluate the viability of our BTO on LSAT as a new photonic platform, we fabricated a Mach–Zehnder interferometer (MZI) modulator featuring two 50:50 Y-branch splitters and a pair of 1-mm-long phase-shifting arms (Fig. 4a). One arm incorporates a coplanar waveguide traveling-wave electrode in a ground–signal–ground (GSG) layout, delivering both a radio-frequency signal and a tunable DC bias (see Supplementary Information Note 7). This bias stabilizes the ferroelectric polarization of the BTO layer, ensuring a robust electro-optic response. The other arm employs a ground–signal (GS) capacitive electrode driven by a low DC bias, which induces a fine phase shift to tune the optical operating point.

Figure 4b shows the cross-sectional view of the modulator, where Si_3_N_4_ strip-loaded waveguides confine light within the BTO layer. Finite-element simulations (Fig. 4c) confirm that the waveguide supports a single transverse electric (TE) mode, with the optical field predominantly confined to the 300-nm-thick BTO film (see Supplementary Information Note 8). The electrodes spaced 5.5 μm apart generate a nearly uniform in-plane electric field across the active layer, achieving a strong electric-optic field overlap. Based on the electro-optic tensor of BTO (Supplementary Information Note 4), only the TE mode is effectively modulated under an applied field along the <110> crystallographic direction. The electric-optic field overlap factor is calculated to be 45%, corresponding to a theoretical half-wave voltage–length product (*V*_π_*L*) of 0.73 V cm. The low-frequency modulation response at 1550 nm (Fig. 4d) yields a half-wave voltage of 7 V. This corresponds to a *V*_π_*L* of 0.7 V cm, in close agreement with the simulated value. In comparison with previously reported BTO-based MZI modulators fabricated on other substrates, where the *V*_π_*L* values were either directly measured^47–49^ or calculated from wavelength shift^8,32,50^, our device demonstrates solidly competitive performance.

Figure 4e further illustrates the electro-optic bandwidth of the device. The frequency response of EO *S*_21_ remains relatively flat, with a 3 dB bandwidth of approximately 12 GHz. A clear roll-off is observed around 12 GHz, primarily due to the lack of group velocity matching between the optical and electrical signals. The group velocity mismatch can be mitigated through further optimization of the electrode and the optical waveguide structures. Nevertheless, the device exhibits a substantially larger EO bandwidth compared to previously reported BTO MZI demonstrations^8,49,50^. Additionally, a relatively flat response is observed between 15 GHz and 28 GHz, with a 6 dB EO bandwidth located at 28 GHz, approaching the best current devices with a reported 6 dB bandwidth of 40 GHz^48^. The reflection coefficient *S*_11_ fluctuates around −15 dB, indicating a good impedance matching. In conclusion, we present the first integration of a BTO-based EO modulator on an LSAT insulating substrate. This device demonstrates both low driving voltage and broad operational bandwidth, underscoring the potential of the oxide-integrated BTO platform for high-speed photonic system applications.

## Discussion

In summary, we have demonstrated a heterogenous buffer-free, strain-engineered strategy for the epitaxial growth of BTO films on oxide insulator substrates, effectively addressing the trade-off between optical confinement and lattice compatibility for photonic integration of ferroelectric thin films. The formation of a self-buffer layer induces lateral strain modulation, stabilizing periodic *a*/*c* domain configurations and orthorhombic phase nanoregions. This structural modulation significantly enhances both the electro-optic response and thermal stability, yielding a Pockels coefficient *r*_42_ exceeding 358 pm V⁻¹ and a Curie temperature of 200 °C. Based on this material platform, we present the first integrated MZI modulator employing epitaxial BTO on LSAT, achieving a low *V*_π_*L* of 0.7 V cm and an electro-optic bandwidth of 28 GHz. These results provide new insights into electro-optic film design and establish a practical route toward high-performance modulators using flexible and cost-effective fabrication processes in integrated photonics.

## Materials and methods

### Sample preparation

Epitaxial BTO films were deposited on (001) LSAT substrates via pulsed laser deposition (KrF excimer laser, 248 nm). For the primary deposition condition, the film was deposited at 680 °C in an oxygen pressure of 5 Pa, at a laser repetition rate of 3 Hz and laser fluence of 1.2 J cm^−2^. Under modified conditions, the substrate temperature and oxygen partial pressure were varied within the ranges of 650–700 °C and 5–10 Pa, respectively, while monitoring reflection high-energy electron diffraction (RHEED, STAIB Instruments) to ensure clear diffraction spots and layer-by-layer oscillations under constant laser energy and frequency. Following the initial 40 nm of growth, the temperature and oxygen pressure were stabilized at 660 °C and 10 Pa, respectively, for the remainder of the deposition. After deposition, all films were annealed in situ for 10 min under an oxygen pressure of 20 kPa.

### STEM

The STEM images and the 4D datasets were acquired on a probe-aberration-corrected FEI Titan Cubed Themis G2 microscope operated at 300 kV. 4D datasets were acquired with a pixel array detector EMPAD. The convergence semi-angle was set to 0.83 mrad. Each diffraction pattern has a dimension of 128 × 128 pixels, and the camera length is 360 mm, giving a reciprocal pixel size of 0.043 Å^−1^.

### Fabrication of BTO-on-LSAT photonic integrated circuit

A 260 nm-thick Si_3_N_4_ layer was first deposited over the BTO film on LSAT substrate by plasma-enhanced chemical vapor deposition (PECVD) to serve as the core material for the subsequent strip waveguide. The photonic waveguide pattern was defined by an electron-beam lithography system (EBPG 5200, Raith). Fluorine-based reactive ion etching (RIE) was employed to form the Si_3_N_4_ waveguides using the electron-beam resist as the etch mask. After waveguide fabrication, a >2 µm-thick patterned optical isolation layer was formed using benzocyclobutene (BCB) photoresist. The electrode patterns were subsequently defined by a UV laser direct-write lithography system (MicroWriter ML3, Durham Magneto Optics), followed by e-beam evaporation of a 10 nm Ti adhesion layer and a 500 nm Au layer. Lift-off was performed to obtain the final metal electrodes. The sample was then diced and facet-polished to form low-loss edge couplers.

### Electro-optic modulator characterization

A tunable laser (TSL-570, Santec) operating in the C-band was used for linear electro-optic tuning measurements, with a fiber polarization controller employed to ensure excitation of the TE mode. For *V*_π_ measurements, the MZI modulator was driven by a 100 kHz triangular voltage signal while monitoring the optical transmission in real time. Electro-optic bandwidth measurements were conducted using a 67 GHz vector network analyzer (PNA N5247B, Keysight) in conjunction with a Bias-T, which enabled application of a DC bias but limited the measurable frequency range up to 40 GHz. A pair of high-speed microwave probes delivered the RF signal to the input of the transmission line, and the output was terminated with a 50 Ω load. Optical coupling into and out of the chip was achieved via lensed fibers. The modulated optical signal was amplified by an erbium-doped fiber amplifier and subsequently detected by a high-speed photodiode, enabling extraction of the *S*_21_ response.

## Supplementary information

Supplementary Information for Self-Buffered Epitaxy of Barium Titanate on Oxide Insulators Enables High-Performance Electro-Optic Modulators

## Data Availability

All data needed to evaluate the conclusions in the paper are present in the paper and/or the Supplementary Information. Additional data can be provided by the authors upon reasonable request.

## References

[1] Shastri, B. J. et al. Photonics for artificial intelligence and neuromorphic computing. *Nat. Photonics* **15**, 102–114 (2021).

[2] Marpaung, D., Yao, J. P. & Capmany, J. Integrated microwave photonics. *Nat. Photonics* **13**, 80–90 (2019).

[3] Hu, Y. W. et al. Integrated electro-optics on thin-film lithium niobate. *Nat. Rev. Phys.* **7**, 237–254 (2025).

[4] Atabaki, A. H. et al. Integrating photonics with silicon nanoelectronics for the next generation of systems on a chip. *Nature* **556**, 349–354 (2018). 29670262 10.1038/s41586-018-0028-z

[5] Wang, C. et al. Integrated lithium niobate electro-optic modulators operating at CMOS-compatible voltages. *Nature* **562**, 101–104 (2018). 30250251 10.1038/s41586-018-0551-y

[6] Yu, M. J. et al. Integrated femtosecond pulse generator on thin-film lithium niobate. *Nature* **612**, 252–258 (2022). 36385531 10.1038/s41586-022-05345-1

[7] Zhu, D. et al. Integrated photonics on thin-film lithium niobate. *Adv. Opt. Photonics* **13**, 242–352 (2021).

[8] Xiong, C. et al. Active silicon integrated nanophotonics: ferroelectric BaTiO_3_ devices. *Nano Lett.* **14**, 1419–1425 (2014). 24447145 10.1021/nl404513p

[9] Wang, H. et al. Advancing inorganic electro-optical materials for 5 G communications: from fundamental mechanisms to future perspectives. *Light Sci. Appl.* **14**, 190 (2025). 40350464 10.1038/s41377-025-01851-9 PMC12066740

[10] Eltes, F. et al. An integrated optical modulator operating at cryogenic temperatures. *Nat. Mater.* **19**, 1164–1168 (2020). 32632281 10.1038/s41563-020-0725-5

[11] Geler-Kremer, J. et al. A ferroelectric multilevel non-volatile photonic phase shifter. *Nat. Photonics* **16**, 491–497 (2022).

[12] Abel, S. et al. A strong electro-optically active lead-free ferroelectric integrated on silicon. *Nat. Commun.* **4**, 1671 (2013). 23575675 10.1038/ncomms2695

[13] Abel, S. et al. Large Pockels effect in micro- and nanostructured barium titanate integrated on silicon. *Nat. Mater.* **18**, 42–47 (2019). 30420671 10.1038/s41563-018-0208-0

[14] Petraru, A. et al. Ferroelectric BaTiO_3_ thin-film optical waveguide modulators. *Appl. Phys. Lett.* **81**, 1375–1377 (2002).

[15] Wessels, B. W. Ferroelectric epitaxial thin films for integrated optics. *Annu. Rev. Mater. Res.* **37**, 659–679 (2007).

[16] Kim, I. D. et al. Ridge waveguide using highly oriented BaTiO_3_ thin films for electro-optic application. *J. Asian Ceram. Societ.* **2**, 231–234 (2014).

[17] Cao, Y. et al. A barium titanate-on-oxide insulator optoelectronics platform. *Adv. Mater.* **33**, 2101128 (2021). 10.1002/adma.202101128 34323320

[18] Lee, J. W. et al. In-plane quasi-single-domain BaTiO_3_ via interfacial symmetry engineering. *Nat. Commun.* **12**, 6784 (2021). 34811372 10.1038/s41467-021-26660-7 PMC8608839

[19] Wang, T. Q. et al. Critical thickness and strain relaxation in molecular beam epitaxy-grown SrTiO_3_ films. *Appl. Phys. Lett.* **103**, 212904 (2013).

[20] Yu, H. et al. Tuning the electro-optic properties of BaTiO_3_ epitaxial thin films via buffer layer-controlled polarization rotation paths. *Adv. Funct. Mater.* **34**, 2315579 (2024).

[21] Huangfu, G. et al. Giant electric field–induced strain in lead-free piezoceramics. *Science* **378**, 1125–1130 (2022). 36480626 10.1126/science.ade2964

[22] Liu, Q. et al. Practical high-performance lead-free piezoelectrics: structural flexibility beyond utilizing multiphase coexistence. *Natl Sci. Rev.* **7**, 355–365 (2020). 34692051 10.1093/nsr/nwz167 PMC8288886

[23] Lv, X. et al. Emerging new phase boundary in potassium sodium-niobate based ceramics. *Chem. Soc. Rev.* **49**, 671–707 (2020). 31913391 10.1039/c9cs00432g

[24] Liu, H. J. et al. Giant piezoelectricity in oxide thin films with nanopillar structure. *Science* **369**, 292–297 (2020). 32675370 10.1126/science.abb3209

[25] Li, W. T., Landis, C. M. & Demkov, A. A. Domain morphology and electro-optic effect in Si-integrated epitaxial BaTiO_3_ films. *Phys. Rev. Mater.* **6**, 095203 (2022).

[26] Jiang, Y. et al. Enabling ultra-low-voltage switching in BaTiO_3_. *Nat. Mater.* **21**, 779–785 (2022). 35618823 10.1038/s41563-022-01266-6

[27] Reitze, D. H. et al. Electro-optic properties of single crystalline ferroelectric thin films. *Appl. Phys. Lett.* **63**, 596–598 (1993).

[28] Dubourdieu, C. et al. Switching of ferroelectric polarization in epitaxial BaTiO_3_ films on silicon without a conducting bottom electrode. *Nat. Nanotechnol.* **8**, 748–754 (2013). 24077030 10.1038/nnano.2013.192

[29] Liu, Q. et al. High-performance lead-free piezoelectrics with local structural heterogeneity. *Energy Environ. Sci.* **11**, 3531–3539 (2018).

[30] Zgonik, M. et al. Dielectric, elastic, piezoelectric, electro-optic, and elasto-optic tensors of BaTiO_3_ crystals. *Phys. Rev. B* **50**, 5941–5949 (1994). 10.1103/physrevb.50.5941 9976963

[31] Bernasconi, P., Zgonik, M. & Günter, P. Temperature dependence and dispersion of electro-optic and elasto-optic effect in perovskite crystals. *J. Appl. Phys.* **78**, 2651–2658 (1995).

[32] Posadas, A. B. et al. Thick BaTiO_3_ epitaxial films integrated on Si by RF sputtering for electro-optic modulators in Si photonics. *ACS Appl. Mater. Interfaces* **13**, 51230–51244 (2021). 34669388 10.1021/acsami.1c14048

[33] Chelladurai, D. et al. Barium titanate and lithium niobate permittivity and Pockels coefficients from megahertz to sub-terahertz frequencies. *Nat. Mater.* **24**, 868–875 (2025). 40097599 10.1038/s41563-025-02158-1 PMC12133585

[34] Edmondson, B. I. et al. Epitaxial, electro-optically active barium titanate thin films on silicon by chemical solution deposition. *J. Am. Ceram. Soc.* **103**, 1209–1218 (2020).

[35] Reynaud, M. et al. Electro-optic response in epitaxially stabilized orthorhombic *mm*2 BaTiO_3_. *Phys. Rev. Mater.* **5**, 035201 (2021).

[36] Petraru, A. et al. Integrated optical Mach Zehnder modulator based on polycrystalline BaTiO_3_. *Opt. Lett.* **28**, 2527–2529 (2003). 14690136 10.1364/ol.28.002527

[37] Kormondy, K. J. et al. Microstructure and ferroelectricity of BaTiO_3_ thin films on Si for integrated photonics. *Nanotechnology* **28**, 075706 (2017). 27973350 10.1088/1361-6528/aa53c2

[38] Picavet, E. et al. Integration of solution-processed BaTiO_3_ thin films with high Pockels coefficient on photonic platforms. *Adv. Funct. Mater.* **34**, 2403024 (2024).

[39] Deng, C. G. et al. Reporting excellent transverse piezoelectric and electro-optic effects in transparent rhombohedral PMN-PT single crystal by engineered domains. *Adv. Mater.* **33**, 2103013 (2021). 10.1002/adma.202103013 34510568

[40] Liu, X. et al. Ferroelectric crystals with giant electro-optic property enabling ultracompact Q-switches. *Science* **376**, 371–377 (2022). 35446634 10.1126/science.abn7711

[41] Gradauskaite, E. et al. Defeating depolarizing fields with artificial flux closure in ultrathin ferroelectrics. *Nat. Mater.* **22**, 1492–1498 (2023). 37783942 10.1038/s41563-023-01674-2 PMC10713449

[42] Choi, K. J. et al. Enhancement of ferroelectricity in strained BaTiO_3_ thin films. *Science* **306**, 1005–1009 (2004). 15528439 10.1126/science.1103218

[43] Li, W. et al. Delineating complex ferroelectric domain structures *via* second harmonic generation spectral imaging. *J. Mater.* **9**, 395–402 (2023).

[44] Nelson, C. T. et al. Domain dynamics during ferroelectric switching. *Science* **334**, 968–971 (2011). 22096196 10.1126/science.1206980

[45] Chen, Z. B. et al. Facilitation of ferroelectric switching via mechanical manipulation of hierarchical nanoscale domain structures. *Phys. Rev. Lett.* **118**, 017601 (2017). 28106439 10.1103/PhysRevLett.118.017601

[46] Xu, R. J. et al. Ferroelectric polarization reversal via successive ferroelastic transitions. *Nat. Mater.* **14**, 79–86 (2015). 25344784 10.1038/nmat4119

[47] Dong, Z. M. et al. Monolithic barium titanate modulators on silicon-on-insulator substrates. *ACS Photonics* **10**, 4367–4376 (2023).

[48] Li, W. J. et al. Thin-film BTO-based MZMs for next-generation IMDD transceivers beyond 200 Gbps/λ. *J. Lightw. Technol.* **42**, 1143–1150 (2024).

[49] Team, P. si Q. uantum A manufacturable platform for photonic quantum computing. *Nature* **641**, 876–883 (2025). 40010377 10.1038/s41586-025-08820-7 PMC12095036

[50] Eltes, F. et al. A BaTiO_3_-based electro-optic Pockels modulator monolithically integrated on an advanced silicon photonics platform. *J. Lightw. Technol.* **37**, 1456–1462 (2019).

