# Supplementary material for: Self-buffered epitaxy of barium titanate on oxide insulators enables high-performance electro-optic modulators
Source: Light Sci Appl. 2026 Jan 2;15:21. doi: 10.1038/s41377-025-02081-9 (PMC12757598; doi:10.1038/s41377-025-02081-9)
Supplement: Supplementary file 1 — Supplementary Information for Self-Buffered Epitaxy of Barium Titanate on Oxide Insulators Enables High-Performance Electro-Optic Modulators [file 41377_2025_2081_MOESM1_ESM.docx]

**Supplementary Information for Self-Buffered Epitaxy of Barium Titanate on Oxide Insulators Enables High-Performance Electro-Optic Modulators**

Chenguang Deng^1, #^, Yutong He^2, #^, Wenfeng Yang^1, #^, Han Yu^1, #^, Zijian Hong^3,4,5^, Hao Liu^2^, Haojie Han^1^, Wei Li^1^, Yunpeng Ma^1^, Zhongshan Zhang^6^, Yongjun Wu^3,4,5^, Jing Ma^1^, Bing Xiong^2^, Changzheng Sun^2,^*, Rong Yu^1^, Jing-Feng Li^1^, Ji Zhou^1^, Yi Luo^2^, Qian Li^1,^*

^1^ State Key Laboratory of New Ceramic Materials, School of Materials Science and Engineering, Tsinghua University, Beijing 100084, China

^2^ Beijing National Research Centre for Information Science and Technology (BNRist), State Key Laboratory of Space Network and Communications, Department of Electronic Engineering, Tsinghua University, Beijing 100084, China

^3^ State Key Laboratory of Silicon and Advanced Semiconductor Materials, School of Materials Science and Engineering, Zhejiang University, Hangzhou, Zhejiang 310058, China

^4^ Zhejiang Key Laboratory of Advanced Solid State Energy Storage Technology and Applications, Taizhou Institute of Zhejiang University, Taizhou, Zhejiang 318000, China

^5^ Institute of Fundamental and Transdisciplinary Research, Zhejiang University, Hangzhou 310058, China

^6^ Beijing National Laboratory for Condensed Matter Physics, Institute of Physics, Chinese Academy of Sciences, Beijing, 100190 China

^#^Equally contributed to this work

* **Corresponding author.**

E-mail: [czsun@tsinghua.edu.cn](mailto:czsun@tsinghua.edu.cn) (C.-Z.S.); [qianli_mse@tsinghua.edu.cn](mailto:qianli_mse@tsinghua.edu.cn) (Q.L.)

**This PDF file includes:**

**Supplementary Notes 1 to 8**

**Figs. S1 to S7**

**Supplementary Notes**

**Note 1. Comparison of Electro-Optic Properties of BaTiO_3_ Thin Films**

A literature review was conducted on the electro-optic (EO) properties of BaTiO_3_ (BTO) thin films synthesized using various techniques, including RF magnetron sputtering (RFMS)^1–3^, molecular beam epitaxy (MBE)^4,5^, pulsed laser deposition (PLD)^6,7^, sol-gel^8,9^, and metal-organic chemical vapor deposition (MOCVD)^10^, with substrates compatible with integrated photonics, such as MgO, Silicon-on-insulator (SOI), (LaAlO_3_)_0.3_-(Sr_2_TaAlO_6_)_0.7_ (LSAT), and oxide buffer layers including SrTiO_3_ (STO) and BaSnO_3_ (BSO). Classical studies have reported EO coefficients for bulk BTO, with unclamped and clamped values of approximately 1000 pm V⁻¹ and 560 pm V⁻¹, respectively, near the telecommunication wavelength (∼1500 nm)^11,12^.In this work, we adopt the clamped value as a practical benchmark, as high-frequency operation and epitaxial stress in integrated photonic platforms make the manifestation of ideal unclamped behavior under in-plane electric fields unlikely.

The comparison of EO coefficients across different studies is complicated by variations in measurement methodologies and the absence of standardized calibration procedures (e.g., using thin-film LiNbO_3_ as a reference). Moreover, the effective EO response of BTO thin films is highly anisotropic, depending on measurement geometry, light propagation direction, and field orientation. Against this backdrop, our work highlights the significant performance gains achieved through structure-driven growth optimization over conventional methods^13^. Our EO coefficient values are calibrated against a standard X-cut LNOI film (300 nm, NANOLN), and the extracted tensor components are consistent with actual device performance. Notably, recent reports show that the *r_42_* coefficient of BTO films on SOI reaches ∼490 pm  V^-1^ at low frequencies and ∼300 pm V^-1^ at frequencies above 100 GHz^3^, with values approaching those of bulk BTO. These findings support more conservative estimates and suggest that reports of exceptionally large EO coefficients should be interpreted within the framework of reproducible and calibrated measurements.

**Note 2. Lattice Distortion and Contrast Analysis in HAADF-STEM Imaging**

Both the low-magnification HAADF-STEM image in Fig. 2a and the high-resolution HAADF-STEM image in Fig. 2e exhibit noticeable contrast in the background. This contrast is not indicative of typical defects or dislocations but rather is attributed to the in-plane stress discussed in the manuscript. This stress results in lattice distortion.

Fig. S3b illustrates the images obtained by varying the defocus. When the electron beam is focused on the center of the sample (left image), the atomic structure shows no noticeable lattice distortion, and the contrast differences between the left and right regions are attributed to lattice distortion. Upon adjusting the defocus to focus the electron beam on the surface of the sample (right image), distinct lattice distortion becomes evident. Due to the depth resolution limitation of the HAADF technique, atoms from different depths within the sample are visible. This distorted lattice region corresponds to the contrast change observed in the left image. By reducing the camera length and collecting electrons scattered at larger angles, the diffraction contrast in the image is minimized. As shown in Fig. S3d, at this setting, the contrast in the image primarily reflects the atomic number and sample thickness. When data is collected with a shorter camera length, as in Fig. S3c, the diffraction contrast caused by lattice distortion, as seen in Fig. S3b, is absent. Additionally, Fig. S3c demonstrates that changing the defocus results in different images corresponding to various depths within the sample.

Energy-dispersive X-ray spectroscopy (EDS) analysis (Fig. S3e) confirms that the contrast observed in HAADF imaging is not related to elemental distribution, which remains uniform. This highlights that the contrast seen in HAADF imaging is independent of defects such as A-site, B-site, or oxygen vacancies.

**Note 3. In situ SHG mapping and electro-optic characterization of domain switching dynamics**

Under in-plane pulsed electric field excitation, BTO domains undergo in-plane reorientation, which can be effectively characterized via normally incident SHG measurements. As shown in Fig. S4, after applying a defined number of pulsed square-wave voltages along the <110> direction, SHG mapping—with both the polarizer and analyzer aligned parallel to <110> —reveals strong contrast in signal intensity between different regions of the BTO film. SHG polarimetry was subsequently performed at two representative points. Point 1, which shows higher SHG intensity, exhibits a distinct twofold symmetry with maximum signal along 45°. In contrast, point 2 displays a weak fourfold symmetry and significantly reduced overall intensity. The twofold pattern at point 1 is consistent with a 1:1 mixture of *a*_1_ and *a*_2_ domains, where the net in-plane polarization is oriented along <110> (i.e., 45°). Such domain superposition gives rise to constructive interference in the SHG response along the polarization axis. A pure in-plane domain state, including *a*_1_, *a*_2_, −*a*_1_, or −*a*_2_ configurations, is expected to produce a fourfold symmetric SHG response. The weak signal and altered symmetry observed at point 2 can be attributed to the coexistence of multiple in-plane domain variants within the probing area (~500 nm for 800 nm femtosecond laser). The cancellation of antiparallel domain contributions yields a net residual polarization, resulting in an SHG response that resembles that of a single in-plane domain but with markedly reduced intensity. Therefore, the intensity distribution in SHG mapping reflects the spatial arrangement of domains reoriented by the external electric field.

In the pulsed EO measurements, initial domain configurations were established by applying a negative poling field. A defined number of square-wave voltage pulses (100 kHz, 50% duty cycle) was then applied to progressively reorient the domains. The EO signal amplitude reflects the net polarization magnitude, while the phase detected by the lock-in amplifier indicates the direction of polarization relative to the initial state. As shown in Fig. S4b and S4c, the domain dynamics extracted from EO and SHG measurements are consistent in terms of switching times. Notably, the EO signal exhibits a distinct two-stage switching process that cannot be described by conventional Kolmogorov–Avrami–Ishibashi (KAI) or nucleation-limited switching (NLS) models. To clarify this behavior, a moderate DC bias was applied in the main manuscript measurements, which enhances the separation between the two switching stages and enables clearer resolution of their respective dynamics.

**Note 4. Coordinate Transformation of the Electro-optic Coefficient Tensor**

In both our measurements and practical device applications, the electric field is applied along the <110> direction or its symmetry-equivalent axes. To determine the relevant electro-optic response under this configuration, the electro-optic coefficient tensor of the BTO film is rotated from the standard cubic <001> coordinate system. The corresponding rotation matrix is given by:

$a=R_{x}=\left[ \begin{matrix} 1 & 0 & 0 \\ 0 & \cos\theta& -\sin\theta\\ 0 & \sin\theta& \cos\theta\end{matrix} \right]$ (1)

In the <100> _c_ coordinate system, the electro-optic coefficient tensor of *a*-domain BTO is given by:

$r=\left[ \begin{matrix} 0 & 0 & r_{13} \\ 0 & 0 & r_{13} \\ 0 & 0 & r_{33} \\ 0 & r_{42} & 0 \\ r_{42} & 0 & 0 \\ 0 & 0 & 0 \end{matrix} \right]$ (2)

Upon rotation to the new coordinate system aligned with <110>_c_ (i.e., *θ* = 45°), the electro-optic tensor transforms accordingly and satisfies:

$r^{'}=Nra^{T}=N\left[ \begin{matrix} 0 & -\frac{\sqrt{2}}{2}r_{13} & \frac{\sqrt{2}}{2}r_{13} \\ 0 & -\frac{\sqrt{2}}{2}r_{13} & \frac{\sqrt{2}}{2}r_{13} \\ 0 & -\frac{\sqrt{2}}{2}r_{33} & \frac{\sqrt{2}}{2}r_{33} \\ 0 & {\frac{\sqrt{2}}{2}r}_{42} & {\frac{\sqrt{2}}{2}r}_{42} \\ r_{42} & 0 & 0 \\ 0 & 0 & 0 \end{matrix} \right]$ (3)

Here, *N* refers to the Bond matrix, which describes the coordinate transformation of the electro-optic tensor. Accordingly:

$N=\left[ \begin{matrix} 1 & 0 & 0 & 0 & 0 & 0 \\ 0 & \frac{1}{2} & \frac{1}{2} & -\frac{1}{2} & 0 & 0 \\ 0 & \frac{1}{2} & \frac{1}{2} & \frac{1}{2} & 0 & 0 \\ 0 & 1 & -1 & 0 & 0 & 0 \\ 0 & 0 & 0 & 0 & \frac{\sqrt{2}}{2} & \frac{\sqrt{2}}{2} \\ 0 & 0 & 0 & 0 & -\frac{\sqrt{2}}{2} & \frac{\sqrt{2}}{2} \end{matrix} \right]$ (4)

$r'=\left[ \begin{matrix} 0 & -\frac{\sqrt{2}}{2}r_{13} & \frac{\sqrt{2}}{2}r_{13} \\ 0 & -\frac{\sqrt{2}}{4}r_{13}-\frac{\sqrt{2}}{4}r_{33}-\frac{\sqrt{2}}{4}r_{42} & \frac{\sqrt{2}}{4}r_{13}+\frac{\sqrt{2}}{4}r_{33}-\frac{\sqrt{2}}{4}r_{42} \\ 0 & -\frac{\sqrt{2}}{4}r_{13}-\frac{\sqrt{2}}{4}r_{33}+\frac{\sqrt{2}}{4}r_{42} & \frac{\sqrt{2}}{4}r_{13}+\frac{\sqrt{2}}{4}r_{33}+\frac{\sqrt{2}}{4}r_{42} \\ 0 & -{\frac{\sqrt{2}}{2}r}_{13}+{\frac{\sqrt{2}}{2}r}_{33} & {\frac{\sqrt{2}}{2}r}_{13}-{\frac{\sqrt{2}}{2}r}_{33} \\ \frac{\sqrt{2}}{2}r_{42} & 0 & 0 \\ -\frac{\sqrt{2}}{2}r_{42} & 0 & 0 \end{matrix} \right]$ (5)

when the electric field is applied along the <110>_c_ direction in the transformed coordinate system, only the *E_z_* component is nonzero. Accordingly, the refractive index ellipsoid is given by:

$\left( \beta_{1}+\Delta\beta_{1} \right)x_{1}^{2}+\left( \beta_{2}+\Delta\beta_{2} \right)x_{2}^{2}+\left( \beta_{3}+\Delta\beta_{3} \right)x_{3}^{2}+\Delta\beta_{4}x_{2}x_{3}=1$ (6)

where:

$\Delta\beta_{1}=\frac{\sqrt{2}}{2}r_{13}E$ (7)

$\Delta\beta_{2}=(\frac{\sqrt{2}}{4}r_{13}+\frac{\sqrt{2}}{4}r_{33}-\frac{\sqrt{2}}{4}r_{42})E$ (8)

$\Delta\beta_{3}=(\frac{\sqrt{2}}{4}r_{13}+\frac{\sqrt{2}}{4}r_{33}+\frac{\sqrt{2}}{4}r_{42})E$ (9)

$\Delta\beta_{4}=({\frac{\sqrt{2}}{2}r}_{13}-{\frac{\sqrt{2}}{2}r}_{33})E$ (10)

In which $\beta={(\frac{1}{n})}^{2}$.

Since the *r*_42_ component is typically much larger than the *r*_33_ and *r*_31_ components, the influence of the cross term $\Delta\beta_{4}$ is generally neglected in practical calculations. Meanwhile, the refractive index change induced by the electro-optic effect is much smaller than the intrinsic refractive index of the material, this leads to:

$d\left( \frac{1}{n^{2}} \right)=-\frac{2}{n^{3}}dn$(11)

$dn=-\frac{1}{2}n^{3}d\left( \frac{1}{n^{2}} \right)=-\frac{1}{2}n^{3}\Delta\beta$ (12)

Therefore, in EO measurements, when the light propagation direction is along the <001> direction and the incident polarization is oriented at 45° with respect to the applied electric field direction, the incident light can be decomposed into polarization components along the *z* and *y* axes of the crystal coordinate system. The refractive indices along these two directions are then given by:

$n_{z}=n_{3}+dn_{3}=n_{3}-\frac{1}{2}{n_{3}}^{3}\Delta\beta_{3}$ (13)

$n_{y}=n_{2}+dn_{2}=n_{2}-\frac{1}{2}{n_{2}}^{3}\Delta\beta_{2}$ (14)

Accordingly, the difference in refractive indices is given by:

$\Delta n=n_{z}-n_{y}=({n_{3}-n}_{2})-\frac{1}{2}{(n_{3}}^{3}\Delta\beta_{3}-{n_{2}}^{3}\Delta\beta_{2})$ (15)

For crystals with negligible natural birefringence, such as barium titanate, the refractive index difference between *n₃* and *n₂* is very small—typically on the order of 0.01. Therefore, it is reasonable to approximate *n₃ ≈ n₂ ≈ n*.

Therefore, the effective electro-optic coefficient is given by:

$\Delta n=-\frac{1}{2}n^{3}r_{c}E$ (16)

$r_{c}=\frac{\Delta\beta_{3}-\Delta\beta_{2}}{E}={\frac{\sqrt{2}}{2}r}_{42}$ (17)

In actual EO devices, the applied electric field is oriented along the <110> direction. In this configuration, only the TE mode exhibits a strong electro-optic response. The polarization of light is aligned with the direction of the applied electric field, corresponding to the *z*-axis in the new coordinate system. Similarly, we obtain:

$r_{c}=\frac{\Delta\beta_{3}}{E}={\frac{\sqrt{2}}{4}r_{13}+\frac{\sqrt{2}}{4}r_{33}+\frac{\sqrt{2}}{4}r}_{42}\approx\frac{\sqrt{2}}{4}r_{42}$ (18)

In the half-wave voltage simulations, an effective electro-optic coefficient of 127 pm V^-1^ was used for the TE mode in the waveguide, corresponding to half the value obtained from our EO measurements.

When the applied electric field is aligned along the in-plane <100> direction, no coordinate transformation is needed to extract the effective electro-optic coefficient *r*_c_ in an *a*-domain BTO sample. Given the negligible natural birefringence, *r*_c_ is independent of the electro-optic tensor element *r*_42_ and can be directly expressed as:

$r_{c}=r_{33}-r_{31}$ (19)

As shown in Fig. 3c of the manuscript, the value of *r*_33_ – *r*_31_ under this configuration is only 34 pm V^-1^, indicating that *r*_33_ and *r*_31_ are substantially smaller than *r*_42_, consistent with the behavior observed in bulk BTO.

**Note 5. Phase-field simulation**

In this study, Phase-field simulations are performed to investigate the polar pattern of the BTO thin film. The spontaneous polarization vector $\vec{P}_{i}$ (*i*=1,3) is selected as the primary order parameter, while the polarization evolution is obtained by solving the time-dependent Ginzburg-Landau equations (TDGL)^14–16^:

$\frac{\partial\vec{P}_{i}}{\partial t}=-L\frac{\delta F}{\delta\vec{P}_{i}} (i=1,3)$ (20)

where *L* is the kinetic coefficient related to the domain wall mobility, *t* is the evolution timestep, *F* is the total free energy which has the contributions from the elastic, electric, Landau, and gradient energies:

$F=\int(f_{elas}+f_{elec}+f_{Land}+f_{grad})dV$ (21)

Detailed expressions of the specific energy densities and the numerical solutions, materials parameters can be found in the literatures^14–18^. A three-dimensional mesh of 200 × 200 × 100 was used, with each grid representing 0.4 nm. Periodic boundary conditions are applied along the *x* and *y* dimensions, while a superposition method is adopted for the out-of-plane direction^19^. The thickness of the substrate, air, and film are set as 30, 50, and 20 grids, respectively. To mimic the local variation in the substrate strain due to the formation of strain, a triangular anisotropic epitaxial strain is applied which varies from -0.8% to 0.4% within half of the simulation in-plane length. Three cases are simulated for different phase angles between strain *ε*_xx_ and *ε*_yy_: i.e., 0°, 90° and 180°, respectively. The normalized timestep is set as 0.01. The total energy and polarization distributions remain consistent across different cases. Fig. S5 illustrates the representative case with a phase angle difference of 0°.

**Note 6. Detailed methods**

**Surface Morphology and Structural Characterization:** Surface morphology was characterized by an atomic force microscopy (AFM, MFP-3D, Asylum Research, USA), which was also operated as a piezoresponse force microscopy (PFM) to conduct measurements. 2*θ*-*ω* XRD scans, rocking curve measurements, and reciprocal space mapping (RSM), was performed using a high-resolution X-ray diffractometer (Empyrean, PANalytical). Temperature-dependent XRD measurements were carried out using the DHS 1100 dome hot stage accessory on the same diffractometer.

**Electro-Optic and SHG Characterization:** For electro-optic and in situ SHG measurements requiring in-plane electrodes, the backsides of the LSAT substrates were polished. Masks of electrode patterns were prepared on the BTO surface by direct laser writing photolithography, followed by platinum deposition via magnetron sputtering. Coplanar electrode pairs were subsequently formed through a lift-off process.

SHG measurements were performed using a home-developed laser-scanning microscope equipped with a Ti:sapphire mode-locked femtosecond laser (MaiTai SP, Spectra-Physics) operating at 800 nm. The linearly polarized excitation beam was directed normally onto the sample through an objective lens (numerical aperture = 0.55). A motorized half-wave plate and a Glan-Taylor polarizer were used to control and analyse the polarization states of the incident and SHG light. In situ SHG imaging was achieved through raster scanning over selected regions via galvanometer mirrors under fixed polarization conditions. Temperature-dependent SHG measurements were carried out using a liquid-nitrogen cryostat (MicrostatHiRes, Oxford Instruments).

The detailed methodology for crystalline phase composition fitting in SHG measurements is described in Ref. [12] and is not repeated here. Notably, the relatively high orthorhombic phase fraction (~40%) observed in Fig. 3e arises from the normal-incidence SHG configuration, which is primarily sensitive to in-plane polarization components. Considering the overall domain configuration of the film, the actual O-phase content is likely lower.

Electro-optic properties were measured using a custom-designed characterization system with a 1550 nm single-mode diode laser (Cnilaser TEM-F1550). The laser beam polarization was conditioned through a Glan–Taylor prism and a motorized half-wave plate, then focused to a spot between the coplanar electrodes using an objective lens. After traversing the sample, the beam passed through a motorized quarter-wave plate and a thin-film polarizer before being collected by an InGaAs detector. During the measurements, the electric field was applied via the coplanar electrodes by superimposing sinusoidal AC modulation signals (10 kHz) and DC offsets. Phase shifts induced by the electro-optic effect were detected by a lock-in amplifier (Zurich Instruments HFLi). Data collected at each test were averaged over 10 seconds using the root mean square (RMS) values to reduce statistical errors.

**Note 7. Electrode Configuration Compatible with Ferroelectric BTO Modulators**

The conventional push–pull configuration typically employed in lithium niobate (LN) modulators was not adopted because of the fundamental difference in coercive fields between LN and BTO thin films. LN exhibits a relatively high coercive field, allowing its electro-optic response to be modulated with a DC bias without altering the polarization state. This enables the two arms of a Mach–Zehnder interferometer (MZI) to operate with opposite RF field directions, creating a 180° phase shift and facilitating efficient intensity modulation, as shown in Fig. S6a.

In contrast, the coercive field of BTO thin films is much lower. Applying a moderate DC bias to tune the optical operating point can easily switch the polarization direction of the BTO layer, aligning both arms with the same effective RF field direction. This results in identical optical phase shifts in both arms, suppressing intensity modulation. Although a pre-poling scheme could, in principle, initialize opposite polarization states in the two arms, such a configuration would preclude the use of DC biasing during operation. Any externally applied field may inadvertently destabilize the preset polarization, leaving wavelength detuning in unbalanced MZIs as the only method for bias control, with the cost of spectral bandwidth.

**Note 8. Characterization of Waveguide Optical Loss Using the Fabry–Pérot (FP) Method**

The loss coefficient can be extracted from the transmission spectrum based on the classical FP formula^20^:

$\alpha L=ln(R\frac{1+\sqrt{\frac{P_{min}}{P_{max}}}}{1-\sqrt{\frac{P_{min}}{P_{max}}}})$ (22)

As shown in Fig. S7a, although the transmission spectrum exhibits certain complexity due to the interference of higher-order modes, the main peak in the Fourier-transformed spectrum matches the calculated free spectral range (FSR), confirming the presence of periodic intensity modulations originating from FP interference. Around 1550 nm, the estimated propagation loss fluctuates around ~7 dB cm^-1^, as shown in Fig. S7b.

**Supplementary Figure**


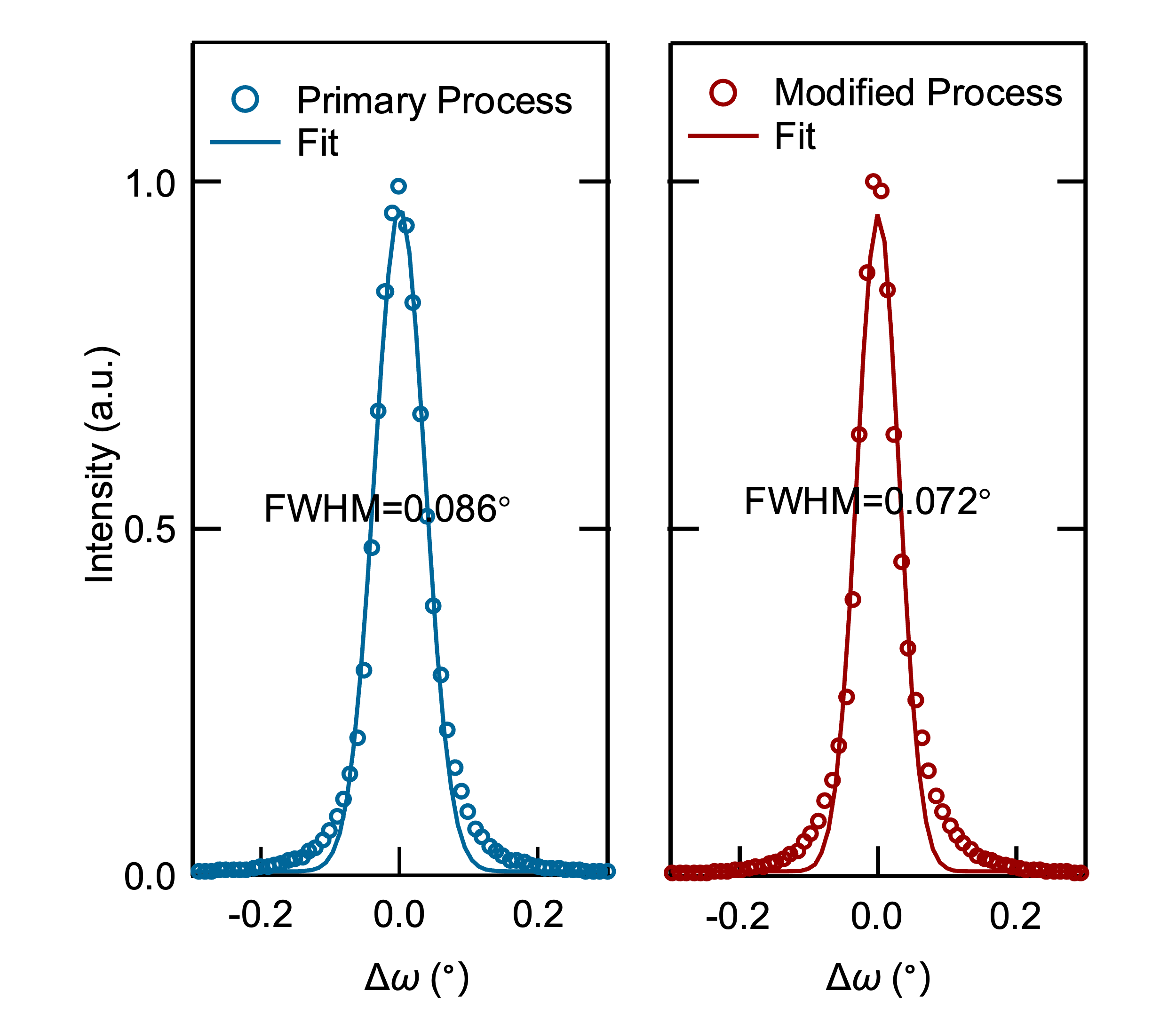


**Fig. S1:** Rocking curve (*ω*-scan) of BTO (002) reflections grown by the primary process and modified process, respectively. The full width at half maximum (FWHM) is indicated in the figure, showing a significantly narrower peak for the Modified Process.


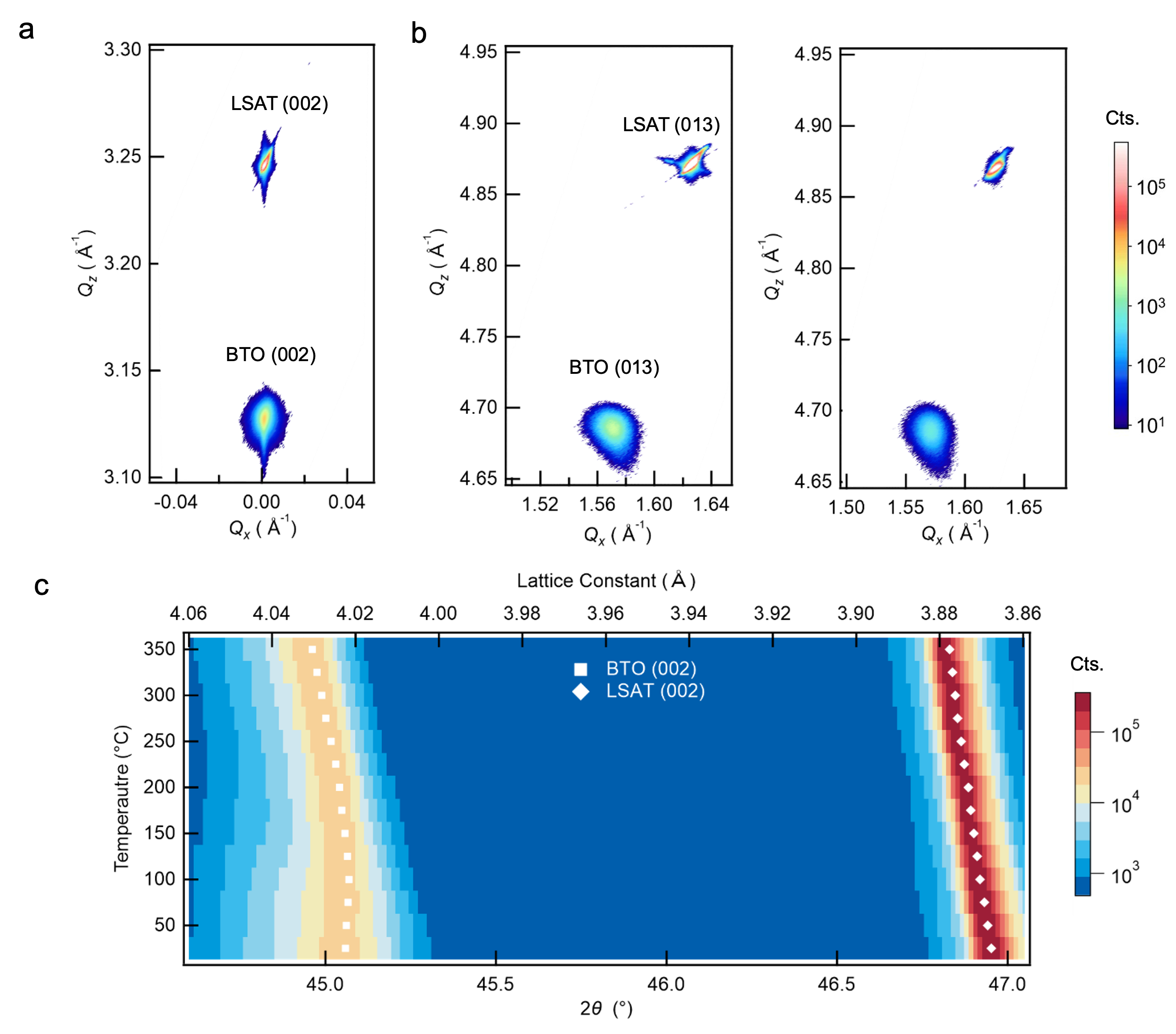


**Fig. S2: a** Reciprocal space mapping (RSM) around the BTO (002) reflection for the sample grown by the modified process. **b** RSMs around the BTO (013) and (103) reflections from the same sample. The near-identical patterns confirm that the in-plane averaged lattice parameters are isotropic across the sample surface. **c** Temperature-dependent 2*θ*–*ω* scans of the BTO (002) reflection. The substrate peak exhibits a linear shift with increasing temperature, consistent with its known thermal expansion coefficient.


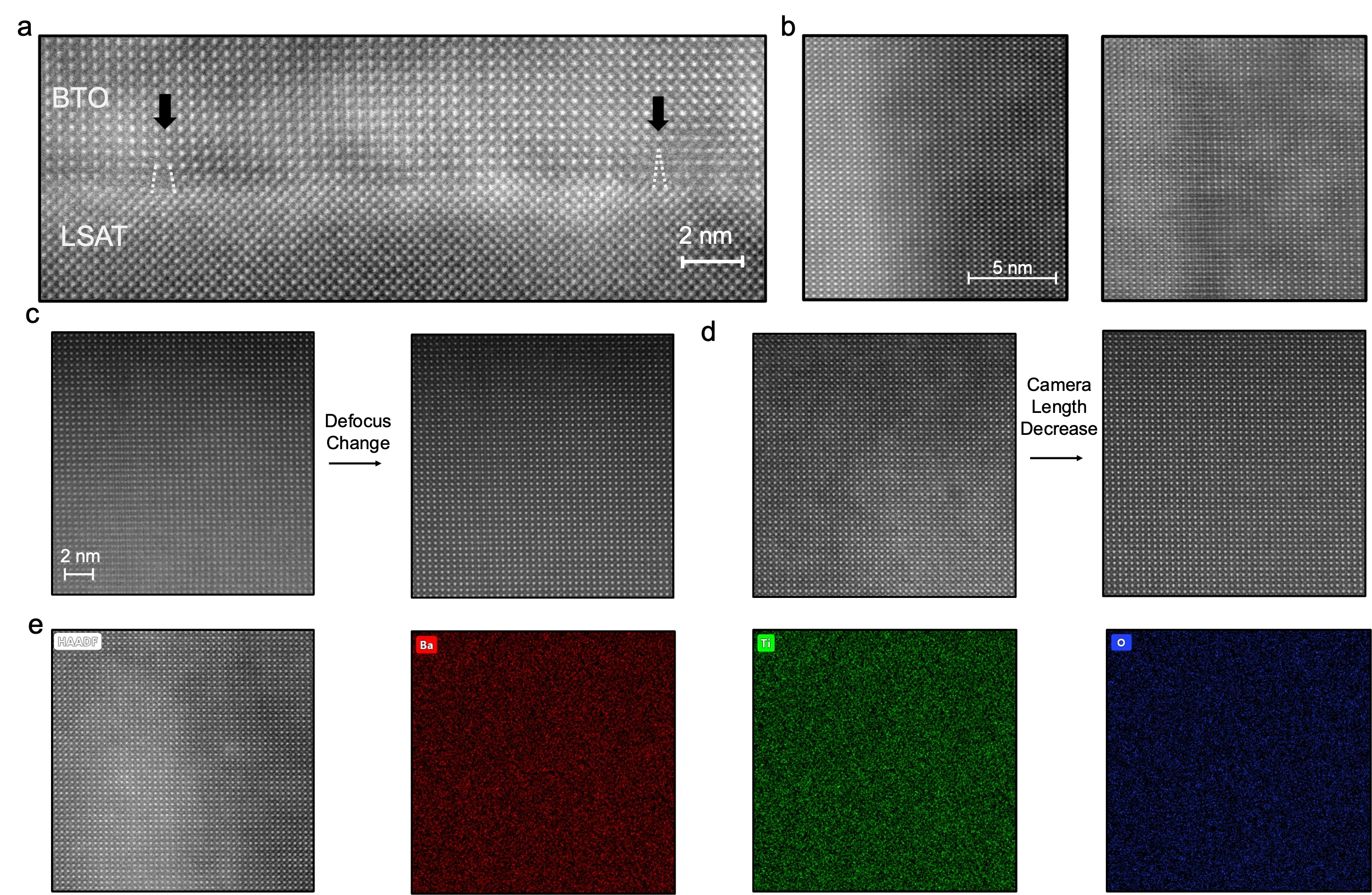


**Fig. S3**: **a** Low-magnification HAADF-STEM image showing a sharp atomic interface between the BTO film and LSAT substrate, the locations of edge dislocations are indicated by black arrows. **b** High-resolution HAADF-STEM images taken under different defocus conditions. Left: when the electron beam is focused on the center of the sample, the lattice appears regular, with contrast variation between left and right regions attributed to in-plane stress–induced lattice distortion. Right: when focused on the surface, pronounced lattice distortion becomes visible, revealing depth-dependent imaging contrast. **c** In certain regions, the atomic contrast appears blurred due to lattice distortion caused by in-plane stress. By adjusting the defocus conditions, these regions can be brought into sharp focus. **d** HAADF-STEM image acquired with reduced camera length, minimizing diffraction contrast and enhancing Z-contrast sensitivity. The absence of stress-induced contrast confirms that the distortion arises from lattice strain rather than compositional variation. **e** Energy-dispersive X-ray spectroscopy (EDS) elemental mapping of the same region, showing a uniform distribution of constituent elements, thereby ruling out contrast contributions from A-site, B-site, or oxygen vacancies.


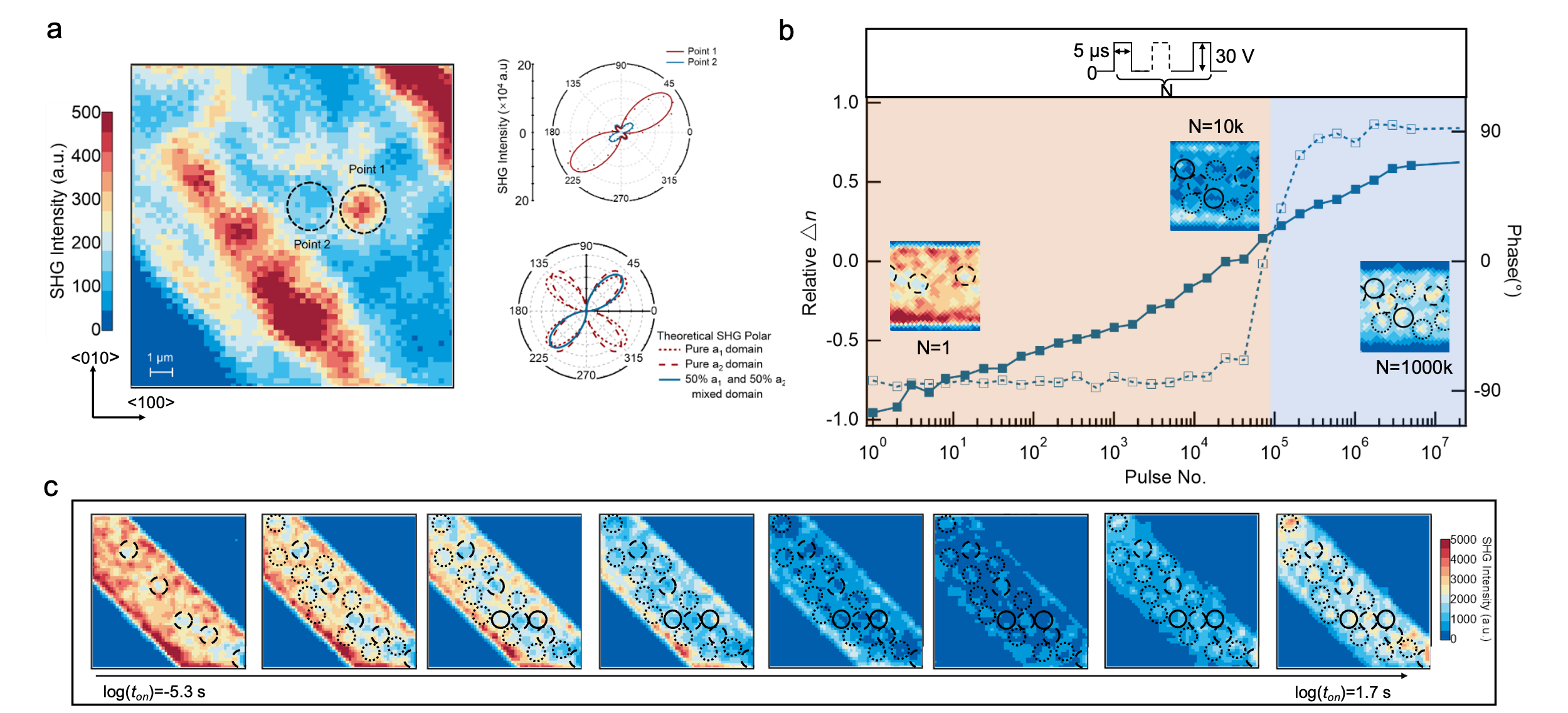


**Fig.** **S4:** **a** SHG mapping of the BTO film under normally incident excitation, with both polarizer and analyzer aligned along the <110> direction. Inset: SHG polarimetry patterns recorded at two representative points. **b** Time-dependent electro-optic (EO) response measured under sequential pulsed electric field excitation. Domain initialization was performed via negative poling. The EO amplitude reflects the net polarization magnitude, and the phase indicates the orientation relative to the initial state. **c** SHG mapping evolution under increasing numbers of pulsed electric field excitations. Each successive image corresponds to a tenfold increase in the number of square-wave voltage pulses compared to the previous one.


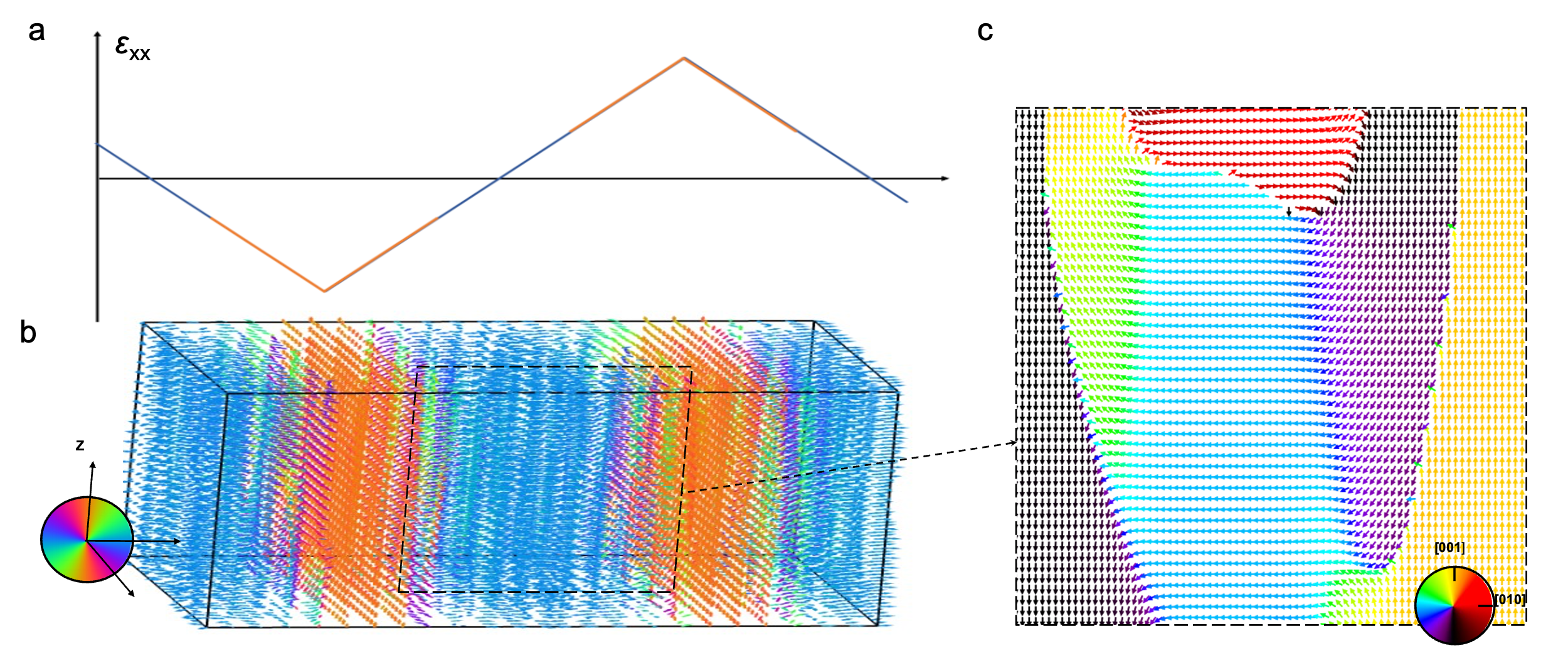


**Fig. S5:** **a** Periodic anisotropic epitaxial strain profile used for simulation, with a phase angle difference of 0° between *ε*_xx_ and *ε*_yy_. **b.c** Phase-field simulation of polarization distribution of modified BTO thin film. **b** three-dimensional distribution of polarization vectors. **c** zoom-in view of cross-sectional polarization vectors in the boxed region of the left figure.


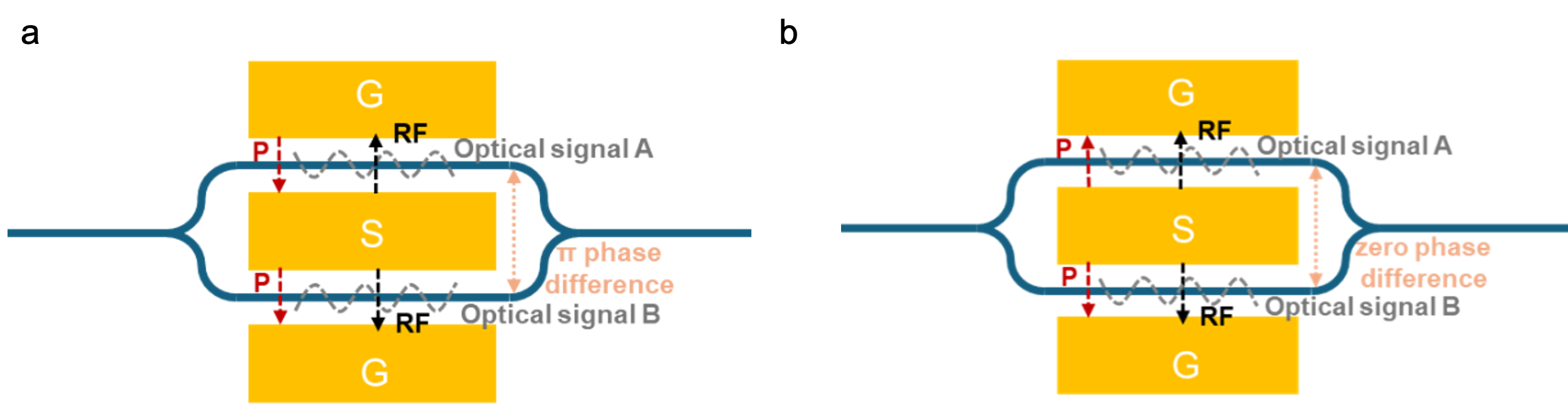


**Fig. S6**: **Push-pull electrode configuration.** **a** In LN thin films, the ferroelectric polarization remains stable regardless of the applied electric field direction. **b** In BTO thin films, the ferroelectric polarization reverses with the polarity of the applied field.


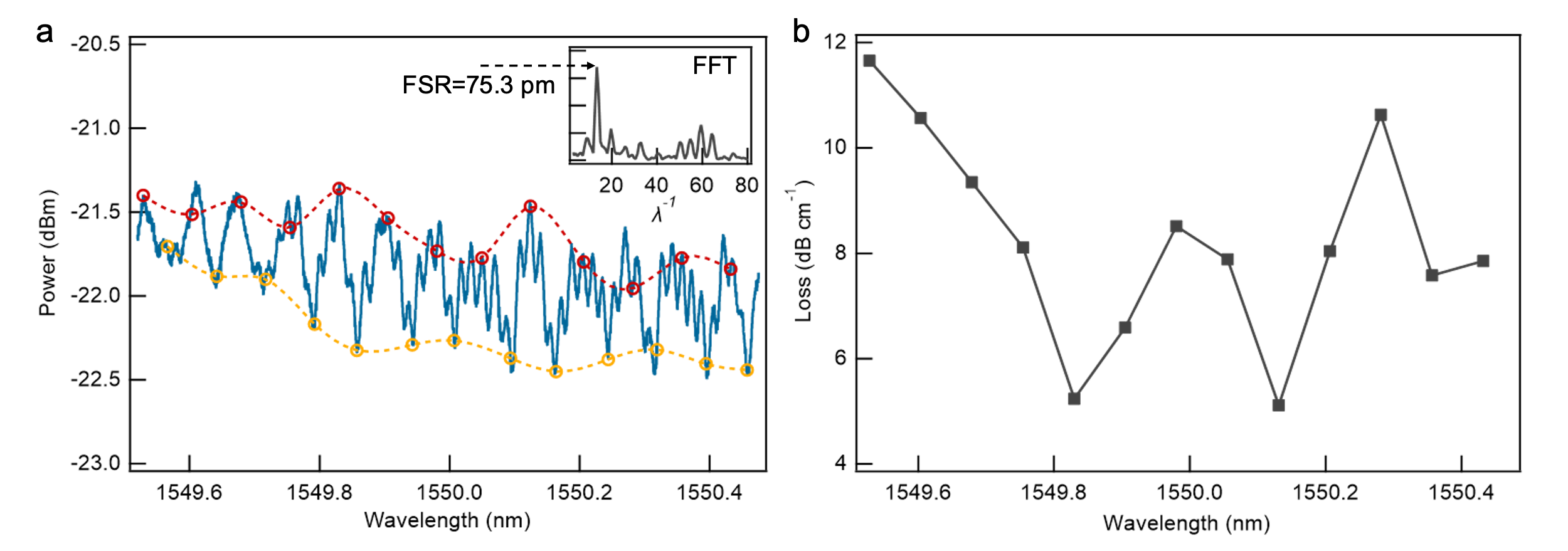


**Fig. S7**: **Characterization of waveguide propagation loss using the Fabry–Pérot method. a** Measured transmission spectrum of a straight waveguide. The inset shows the Fourier transform of the spectrum, from which the free spectral range (FSR) is extracted. **b** Propagation loss coefficient calculated from the periodic interference fringes.

**Supplementary References**

1. Kim, I. D. et al. Ridge waveguide using highly oriented BaTiO_3_ thin films for electro-optic application. *Journal of Asian Ceramic Societies* **2**, 231-234 (2014).

2. Posadas, A. B. et al*.* Thick BaTiO_3_ epitaxial films integrated on Si by RF sputtering for electro-optic modulators in Si photonics. *ACS Applied Materials & Interfaces* **13**, 51230-51244 (2021).

3. Chelladurai, D. et al*.* Barium titanate and lithium niobate permittivity and Pockels coefficients from megahertz to sub-terahertz frequencies. *Nature Materials* **24**, 868-875 (2025).

4. Abel, S. et al*.* A strong electro-optically active lead-free ferroelectric integrated on silicon. *Nature Communications* **4**, 1671 (2013).

5. Reynaud, M. et al*.* Electro-optic response in epitaxially stabilized orthorhombic *mm*2 BaTiO_3_. *Physical Review Materials* **5**, 035201 (2021).

6. Kormondy, K. J. et al*.* Microstructure and ferroelectricity of BaTiO_3_ thin films on Si for integrated photonics. *Nanotechnology* **28**, 075706 (2017).

7. Petraru, A. et al. Integrated optical Mach Zehnder modulator based on polycrystalline BaTiO_3_. *Optics Letters* **28**, 2527-2529 (2003).

8. Edmondson, B. I. et al. Epitaxial, electro‐optically active barium titanate thin films on silicon by chemical solution deposition. *Journal American Ceramic Society* **103**, 1209–1218 (2020).

9. Picavet, E. et al. Integration Of Solution‐Processed BaTiO_3_ Thin Films with High Pockels Coefficient on Photonic Platforms. *Advanced Functional Materials* **34**, 2403024 (2024).

10. Wessels, B. W. Ferroelectric epitaxial thin films for integrated optics. *Annual Review of Materials Research* **37**, 659-679 (2007).

11. Zgonik, M. et al*.* Dielectric, elastic, piezoelectric, electro-optic, and elasto-optic tensors of BaTiO_3_ crystals. *Physical Review B* **50**, 5941-5949 (1994).

12. Bernasconi, P., Zgonik, M. & Günter, P. Temperature dependence and dispersion of electro-optic and elasto-optic effect in perovskite crystals. *Journal of Applied Physics* **78**, 2651-2658 (1995).

13. Yu, H. et al*.* Tuning the electro-optic properties of BaTiO_3_ epitaxial thin films via buffer layer-controlled polarization rotation paths. *Advanced Functional Materials* **34**, 2315579 (2024).

14. Chen, L.-Q. Phase-Field Method of Phase Transitions/Domain Structures in Ferroelectric Thin Films: A Review. *Journal American Ceramic Society* **91**, 1835–1844 (2008).

15. Li, Y. L. et al*.* Effect of substrate constraint on the stability and evolution of ferroelectric domain structures in thin films. *Acta Materialia* **50**, 395–411 (2002).

16. Li, Y. L. et al*.* Effect of electrical boundary conditions on ferroelectric domain structures in thin films. *Applied Physics Letters* **81**, 427–429 (2002).

17. Li, Y. L., Cross, L. E. & Chen, L. Q. A phenomenological thermodynamic potential for BaTiO_3_ single crystals. *Journal of Applied Physics* **98**, 064101 (2005).

18. Tagantsev, A. K. Landau Expansion for Ferroelectrics: Which Variable to Use? *Ferroelectrics* **375**, 19–27 (2008).

19. Chen, L. Q. & Shen, J. Applications of semi-implicit Fourier-spectral method to phase field equations. *Computer Physics Communications* **108**, 147–158 (1998).

20. Tittelbach, G., Richter, B. & Karthe, W. Comparison of three transmission methods for integrated optical waveguide propagation loss measurement. *Pure and Applied Optics: Journal of the European Optical Society Part A* **2**, 683–700 (1993).
